# Separating the rash from the chaff: novel clinical decision support deployed during the mpox outbreak

**DOI:** 10.1017/ice.2024.51

**Published:** 2024-04-02

**Authors:** Jacob E. Lazarus, Chloe V. Green, Michelle S. Jerry, Lindsay Germaine, Dustin S. McEvoy, Caitlin M. Dugdale, Kristen M. Hysell, Rebecca L. Craig, Molly L. Paras, Howard M. Heller, Kevin L. Ard, John S. Albin, Hang Lee, Erica S. Shenoy

**Affiliations:** 1 Division of Infectious Diseases, Department of Medicine, Massachusetts General Hospital, Boston, MA, USA; 2 Harvard Medical School, Boston, MA, USA; 3 Infection Control Unit, Massachusetts General Hospital, Boston, MA, USA; 4 Clinical Informatics and Decision Support, Digital Health, Mass General Brigham, Somerville, MA, USA,; 5 Infection Control, Mass General Brigham, Boston, MA, USA; 6 Biostatistics Center, Massachusetts General Hospital, Boston, MA, USA

## Abstract

A clinical decision support system, EvalMpox, was developed to apply person under investigation (PUI) criteria for patients presenting with rash and to recommend testing for PUIs. Of 668 patients evaluated, an EvalMpox recommendation for testing had a positive predictive value of 35% and a negative predictive value of 99% for a positive mpox test.

## Introduction

Clinical decision support systems (CDSSs) have been shown to increase adherence to clinical guidelines^1^ and augment diagnostic and management behavior in several infectious syndromes.^2–4^ CDSSs assist in diagnosis by allowing correct application of disease-specific criteria, serving as educational tools about unfamiliar syndromes, improving the appropriateness of laboratory testing,^5^ and assisting with the application of isolation precautions.^6^

Emerging infectious diseases place a high cognitive burden on frontline clinicians for several reasons: the clinical presentation is unfamiliar, testing algorithms may change rapidly, detailed epidemiologic history is crucial for identifying at-risk patients, and unfamiliar infection control protocols make applying isolation precautions challenging. The mpox outbreak of 2022–2023 exemplified all these conditions. Since the eradication of smallpox, few clinicians were familiar with poxvirus infections. At the beginning of the epidemic, testing was scarce. And, since infections were concentrated in gay, bisexual, and other men who have sex with men,^7^ it was critical that history taking focus on Centers for Disease Control and Prevention (CDC) epidemiologic criteria for a person under investigation (PUI). These questions, centering on a patient’s sexual health and behaviors, are not universally asked.^8^

To support the identification, isolation, and diagnosis of people presenting with a rash and possible mpox, the “Evaluate for Mpox” (EvalMpox) CDSS was incorporated into the electronic health record (EHR) of a large integrated healthcare system.

## Methods

Based on experience developing CDSS for coronavirus disease 2019 (COVID-19),^6^ a team of infectious diseases, infection control, and information technology experts constructed EvalMpox. Toward quick dissemination at the beginning of the outbreak, we previously communicated a rapid report on the initial CDSS applied to the first 55 evaluated patients ending July 20, 2022.^9^ This manuscript analyzes the performance of an enhanced CDSS and all 668 encounters through April 12, 2023.

**Figure 1.**
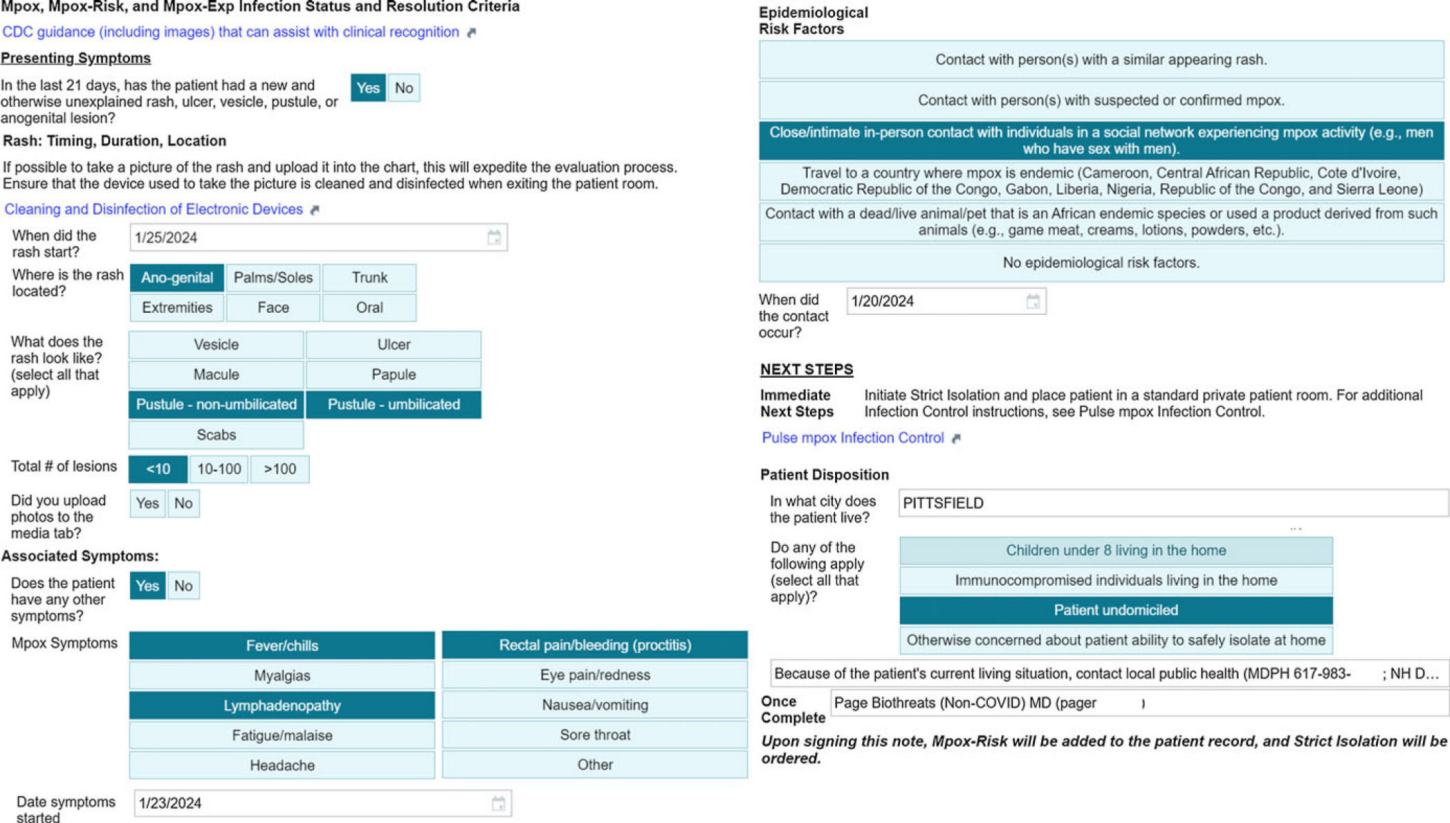
From the top left, EvalMpox guides clinicians to sample images of mpox rashes and guides history taking to allow a standardized collection of information on rash onset, location, qualities, and associated systemic symptoms. It also prompts the clinician to document the rash photographically to assist in the evaluation of rash evolution over time. This standardized approach also accomplishes clinician teaching on features of this unfamiliar disease and ensures evaluation for signs or symptoms that may not be part of a routine evaluation (eg, pharyngitis, proctitis). From the top right, risk factor identification assists with contact tracing. By collecting information on challenges to discharge home, EvalMpox facilitates early involvement of in-house case management and Department of Public Health input. For patients classified as PUI, EvalMpox provides local site contact information to assist HCW in patient triage and testing. Finally, EvalMpox automatically coordinates the application of mpox-related infection statuses and isolation. Example screenshot from Epic^™^ (Epic Systems Corporation). *Note*: HCW, healthcare workers

EvalMpox assists clinicians in identifying patients with mpox by guiding the collection of information regarding epidemiologic criteria for CDC PUI status in patients with a new, unexplained rash (Figure 1). Epidemiologic criteria were updated throughout the epidemic to conform to evolving CDC criteria. If a clinician inputs both clinical and epidemiologic criteria for mpox, EvalMpox classifies the patient as a PUI and recommends testing. If the patient does not meet clinical and epidemiologic criteria for mpox, EvalMpox recommends against testing unless clinical suspicion is high. EvalMpox then generates a risk assessment note in the EHR, coordinates the application of mpox-related infection statuses in the patient’s electronic chart, and orders appropriate isolation (Supplemental Figure 1 online).

Data on EvalMpox encounters exported from the EHR (Epic) included patient demographics, encounter date/time, practice location/setting, clinician-user role, and PUI/non-PUI status. Mpox testing results performed in our system were separately exported. Data were inspected for duplicate encounters, and charts were manually reviewed to ensure data integrity. Categorical data were analyzed with χ^2^ testing and continuous data by *t* test. Negative predictive value (NPV) and positive predictive value (PPV) were calculated over the total analyzed period. Data were collected under MGB IRB protocol 2012P002359.

## Results

### Tool utilization

EvalMpox was used in 668 encounters, originating from over 100 clinical locations across Greater Boston, Nantucket, Martha’s Vineyard, western Massachusetts, and southern New Hampshire (Supplemental Figure 2 online). Encounters originated in the emergency department (n = 219, 33%), urgent care (n = 202, 30%), outpatient (n = 199, 30%), and inpatient (n = 48, 7%) settings (Supplemental Figure 3 online) and peaked in early August 2022 (Supplemental Figure 4 online). EvalMpox was completed by clinicians in diverse role groups, including attending physicians, advanced practice providers, postgraduate trainees, and registered nurses (Supplemental Figure 5 online).

### Patient characteristics

Based on the presence or absence of epidemiologic criteria, EvalMpox classified 275 patients as PUI and 393 patients as non-PUI, respectively. Consistent with national case characteristics reported to the CDC, patients designated PUIs by EvalMpox were significantly younger than those designated non-PUIs (mean age 34 vs 40, *P* value < .001 by *t* test) (Table 1 and Supplemental Figure 6 online). Similarly, PUIs were also significantly more likely to have a recorded legal sex as male (82% vs 55%, *P* < .001 by χ^2^, Table 1 and Supplemental Figure 7 online).

### Mpox testing

PUIs were significantly more likely to be tested for mpox compared with non-PUIs (210 of 275 compared with 53 of 393, *P* < .001 by χ^2^, Table 1 and Supplemental Figure 8 online). Among the tested PUIs, 126 (60%), 74 (35%), and 10 (5%) tested negative, positive, or inconclusive by polymerase chain reaction (PCR), respectively. Among the tested non-PUIs, 49 (92%), 3 (6%), and 1 (2%) tested negative, positive, and inconclusive by PCR, respectively (Table 1 and Supplemental Figure 9 online). Patients designated PUI were significantly more likely to test positive for mpox (*P* < .001 by χ^2^). The PPV of an EvalMpox PUI designation for a positive PCR was 35% (95% CI 29%–42%) and the NPV was 99% (95% CI 98%–100%). One hundred sixteen PCR tests were sent without a corresponding encounter where EvalMpox was performed. Ninety-seven (84%), 13 (11%), and 6 (5%) were negative, positive, and inconclusive by PCR, respectively (Supplemental Figure 10 online).

## Discussion

We describe the performance of EvalMpox, a novel CDSS for the identification, evaluation, and management of patients meeting CDC PUI criteria for mpox. There was widespread adoption of EvalMpox across our large, integrated healthcare system among diverse provider roles and in all care settings. The CDSS performed well; our patients classified as PUI had similar patient demographics compared with CDC mpox case demographics, and PUI were more likely to test positive for mpox than non-PUI. The NPV of EvalMpox was high.

There are several limitations to the conclusions that can be drawn from our report. First, this study was conducted in a single health system, potentially limiting generalizability. Second, though we performed extensive education prior to and during implementation, uptake was not universal. If patient characteristics influenced clinician decisions of whether to use EvalMpox, this utilization behavior may have biased the observed test characteristics of the CDSS. However, EvalMpox was used in most mpox testing encounters. Third, though we find that the NPV for EvalMpox was high, despite this being the largest worldwide mpox outbreak, low overall community prevalence certainly contributes to this result. Fourth, as in any CDSS that relies on provider data entry, errors in tool use can lead to inappropriate recommendations. A focused chart review of the three patients designated non-PUI by EvalMpox who tested positive by PCR revealed that one of those patients reported epidemiologic risk factors for mpox that were not input correctly into EvalMpox.

Finally, clinician judgment remains necessary when interpreting the recommendations from any CDSS. Two individuals who tested positive for mpox reported no epidemiologic risk factors to multiple interviewers and so, following CDC PUI criteria, were designated non-PUI. EvalMpox does direct users to additional clinical resources, and these patients eventually underwent mpox testing due to repeat presentations to care.

In conclusion, our data support the potential for CDSS to assist in the identification, evaluation, and management of patients with emerging infectious diseases, supporting laboratory stewardship and appropriate implementation of transmission-based precautions. Our findings lay the groundwork for future investigations, including into which factors influence healthcare workers (HCW) toward using or not using an available CDSS. It will also be useful, during future outbreaks of emerging infections, to consider randomizing HCW to CDSS use or standard of care, to allow rigorous interrogation of the ability of CDSS to improve diagnostic accuracy and disease-specific knowledge.

## Supplementary Material

Supplementary material

## Figures and Tables

**Table 1.** Characteristics of persons under investigation (PUI) and non-PUI as designated by EvalMpox. One PUI had an unknown legal sex

	PUI (n = 275)	Non-PUI (n = 393)

Age, years (mean, SD)	34 (12)	40 (18)

Female, n (%)	48 (18)	175 (45)

Male, n (%)	226 (82)	218 (55)

PCR tested, n (%)	210 (76)	53 (13)

PCR negative, n (% tested)	126 (60)	49 (92)

PCR positive, n (% tested)	74 (35)	3 (6)

PCR inconclusive, n (% tested)	10 (5)	1 (2)

## References

[1] Sutton RT, Pincock D, Baumgart DC, Sadowski DC, Fedorak RN, Kroeker KI. An overview of clinical decision support systems: benefits, risks, and strategies for success. NPJ Digit Med 2020;3:17. 32047862 10.1038/s41746-020-0221-y PMC7005290

[2] Dezman ZDW, Lemkin D, Papier A, Browne B. The impact of a point-of-care visual clinical decision support tool on admissions for cellulitis in the University of Maryland medical system. J Am Coll Emerg Physicians Open 2023;4:e12969. 10.1002/emp2.12969 PMC10250818 37304858

[3] Williams DJ, Martin JM, Nian H, Antibiotic clinical decision support for pneumonia in the ED: a randomized trial. J Hosp Med 2023;18:491–501. 37042682 10.1002/jhm.13101 PMC10247532

[4] Shaikh N, Hoberman A, Hum SW, Development and validation of a calculator for estimating the probability of urinary tract infection in young febrile children. JAMA Pediatr 2018;172:550–556. 29710324 10.1001/jamapediatrics.2018.0217 PMC6137527

[5] Algaze CA, Wood M, Pageler NM, Sharek PJ, Longhurst CA, Shin AY. Use of a checklist and clinical decision support tool reduces laboratory use and improves cost. Pediatrics. 2016;137:e20143019. 10.1542/peds.2014-3019 26681782

[6] Dugdale CM, Rubins DM, Lee H, COVID-19 diagnostic clinical decision support: a pre-post implementation study of coral (covid risk calculator). Clin Infect Dis 2021;73:2248–2256. 33564833 10.1093/cid/ciab111 PMC7929052

[7] Thornhill JP, Barkati S, Walmsley S, Monkeypox virus infection in humans across 16 countries - April-June 2022. N Engl J Med 2022; 387:679–691. 35866746 10.1056/NEJMoa2207323

[8] Goyal M, McCutcheon M, Hayes K, Mollen C. Sexual history documentation in adolescent emergency department patients. Pediatrics 2011;128:86–91. 21646260 10.1542/peds.2010-1775

[9] Albin JS, Lazarus JE, Hysell KM, Development and implementation of a clinical decision support system tool for the evaluation of suspected monkeypox infection. J Am Med Inform Assoc 2022;29:2124–2127. 36036367 10.1093/jamia/ocac151 PMC9667162

