# Supplementary material for: Separating the rash from the chaff: novel clinical decision support deployed during the mpox outbreak
Source: Infect Control Hosp Epidemiol. Author manuscript; Available in PMC 2025 Jun 18. (PMC11747935; doi:10.1017/ice.2024.51)
Supplement: Supplementary material [file NIHMS2041266-supplement-Supplementary_material.docx]

**Supplemental Figures**


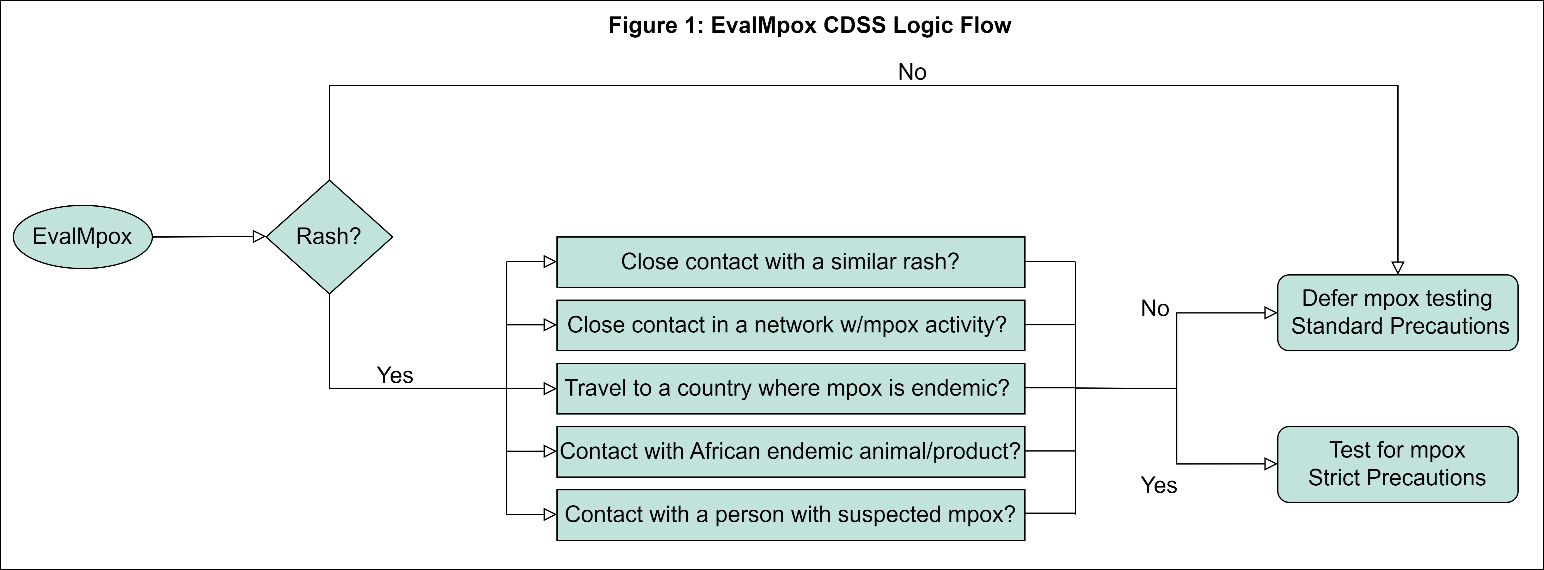


Supplemental Figure 1 - EvalMpox Clinical Decision Support System logic flow. The core function of EvalMpox was to assist in applying CDC person under investigation (PUI) criteria and recommend testing if a PUI, and recommend against testing if not (unless clinical suspicion was high). EvalMpox also automatically coordinated the application of mpox-related infection statuses and isolation.


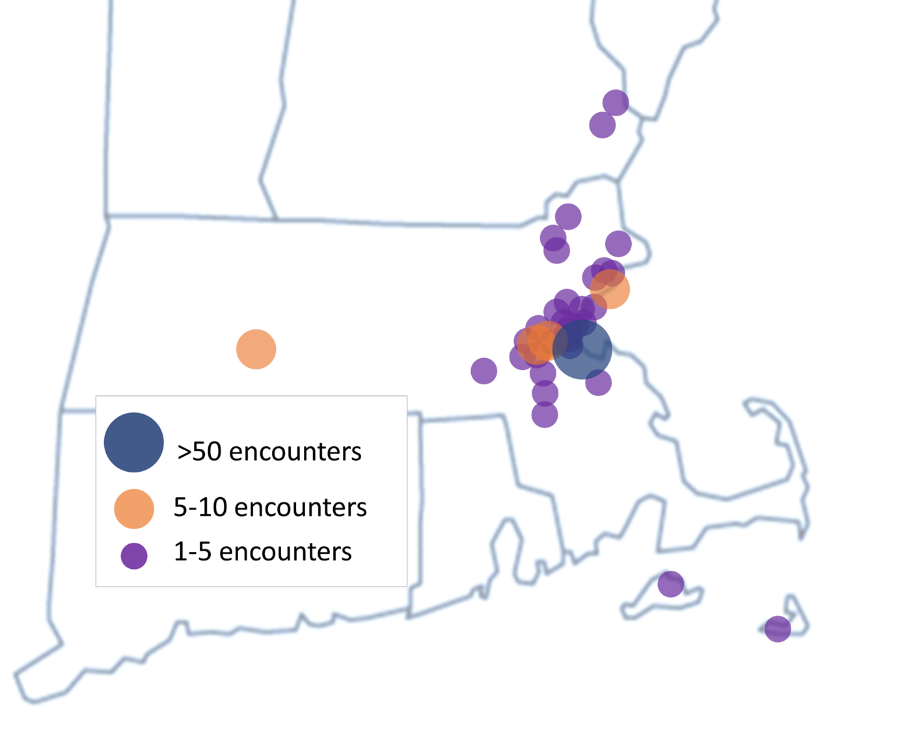


Supplemental Figure 2 - EvalMpox use plotted by practice location. Total encounters within a given municipality are depicted (frequently encompassing multiple individual practices).


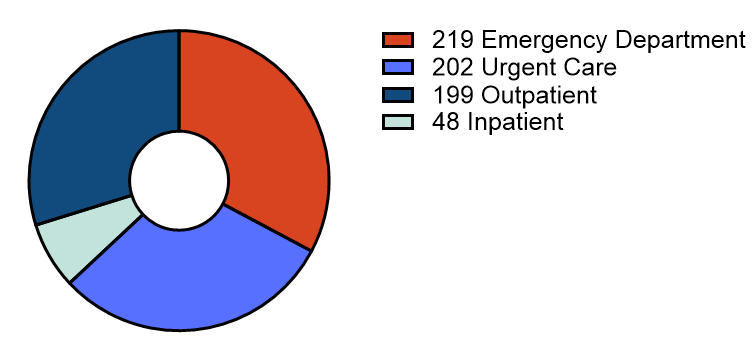


Supplemental Figure 3 - EvalMpox use by care setting. Encounters originated in the emergency department (n=219, 33%), urgent care (n=202, 30%), outpatient (n=199, 30%), and inpatient (n=48, 7%) settings.


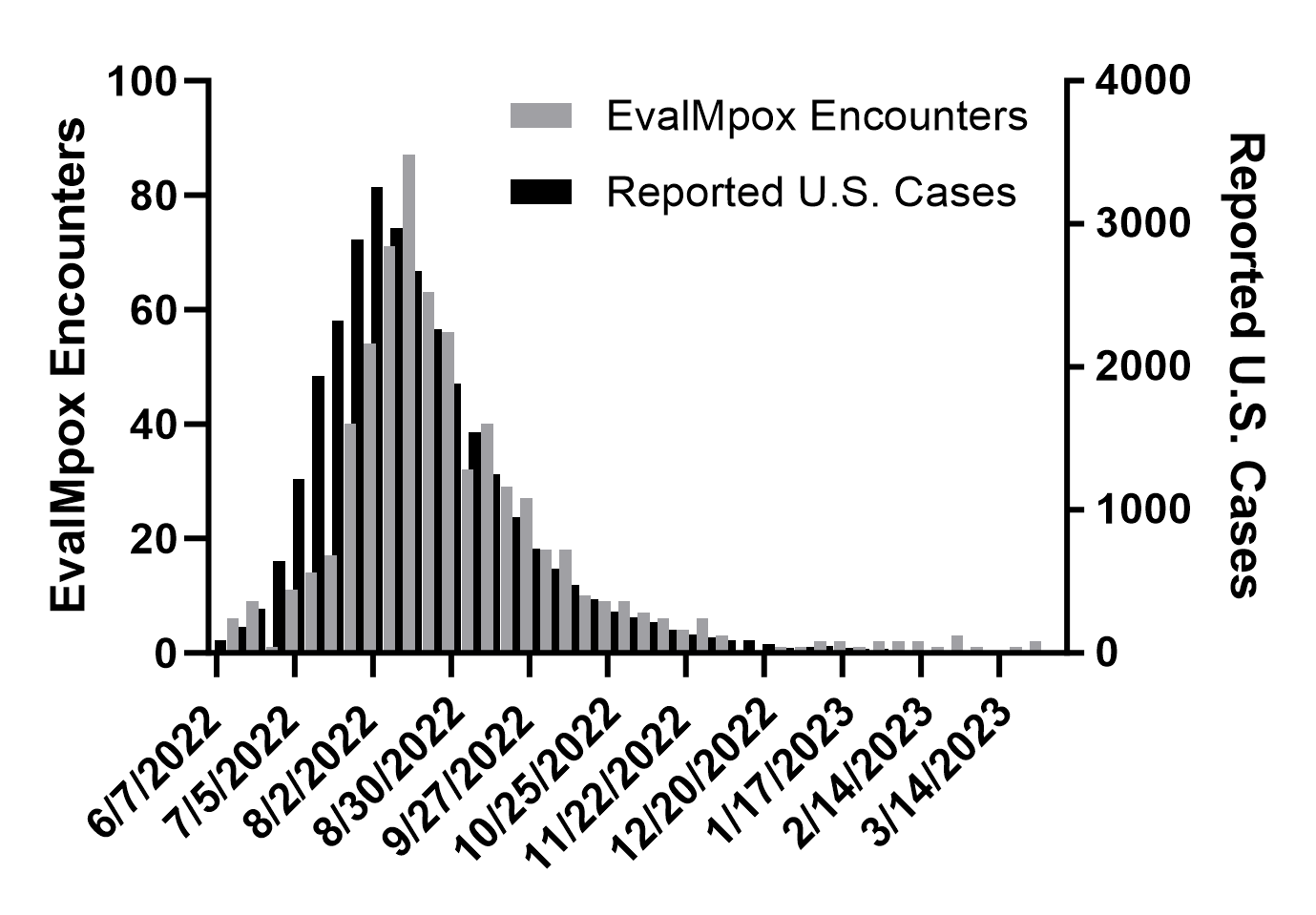


Supplemental Figure 4 – EvalMpox uses, binned by week and depicted in grey, are plotted on the left axis. Reported U.S. cases (CDC), binned by week and depicted in black, are plotted on the right axis. EvalMpox uses track U.S. cases well with an apparent lag in EvalMpox use, perhaps reflecting a slightly delayed epidemic curve in Massachusetts as well as a temporal lag in provider awareness of mpox incidence.


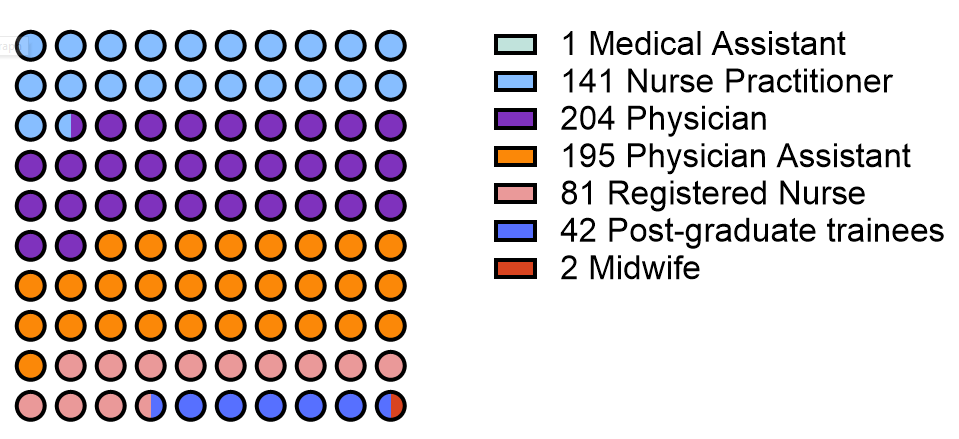


Supplemental Figure 5 – Diverse healthcare personnel utilized EvalMpox, including attending physicians (n-=204, 31%), physician assistants (n=195, 29%), nurse practitioners (n=141, 21%), post-graduate trainees (n=42, 6%), registered nurses (n=81, 12%), and midwives (n=2, 0.3%). 2 encounters had missing role groups.


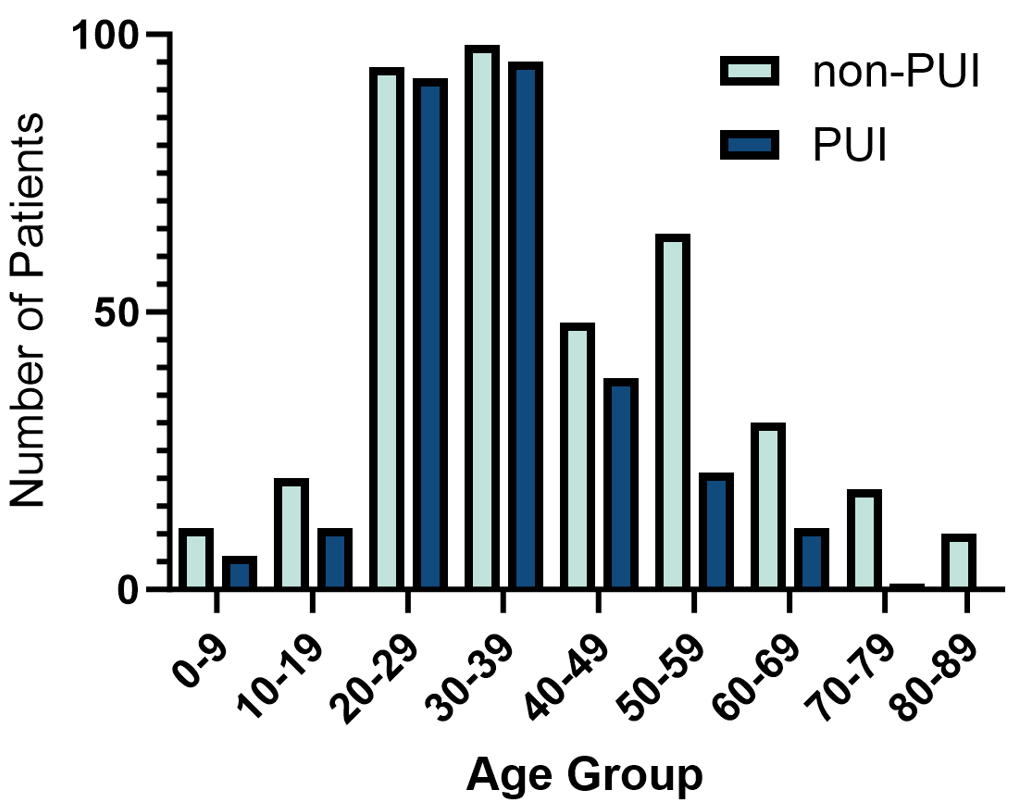


Supplemental Figure 6 – PUI vs. non-PUI age distribution (binned by decade of life). The PUI histogram is roughly normally distributed, centered on ages 20-39, similar to mpox case characteristics. In contrast, a second, minor peak is evident in non-PUIs aged 50-69.


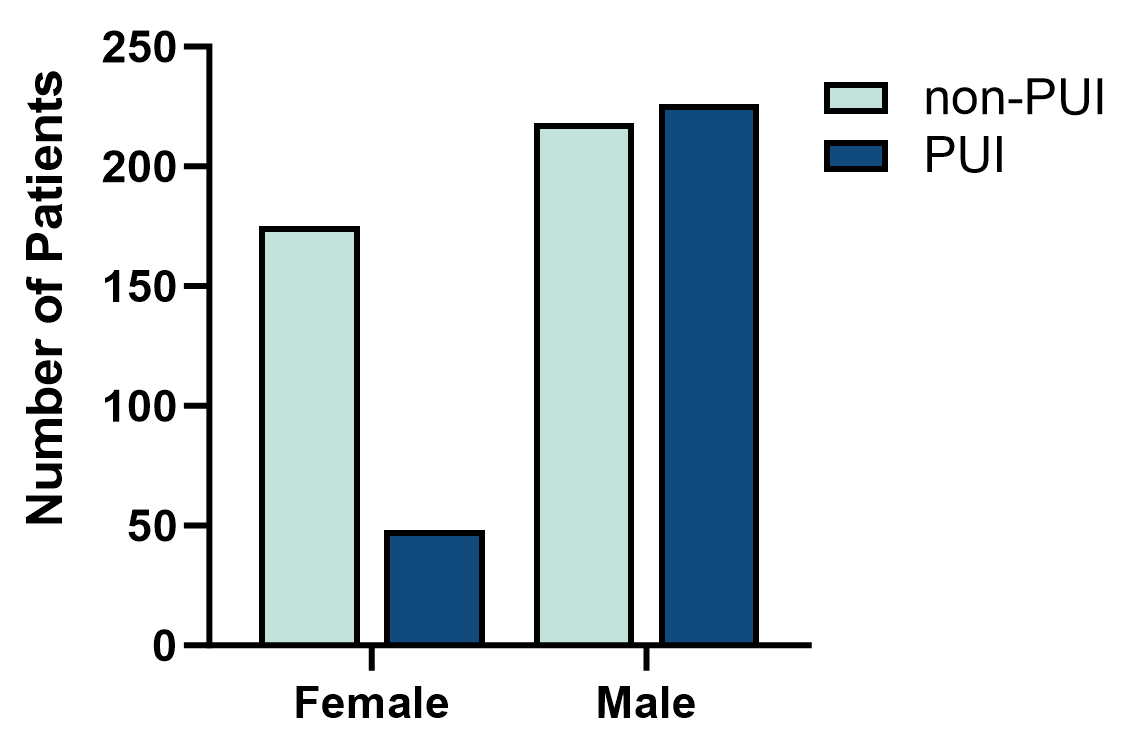


Supplemental Figure 7 – PUI vs. non-PUI reported legal sex.


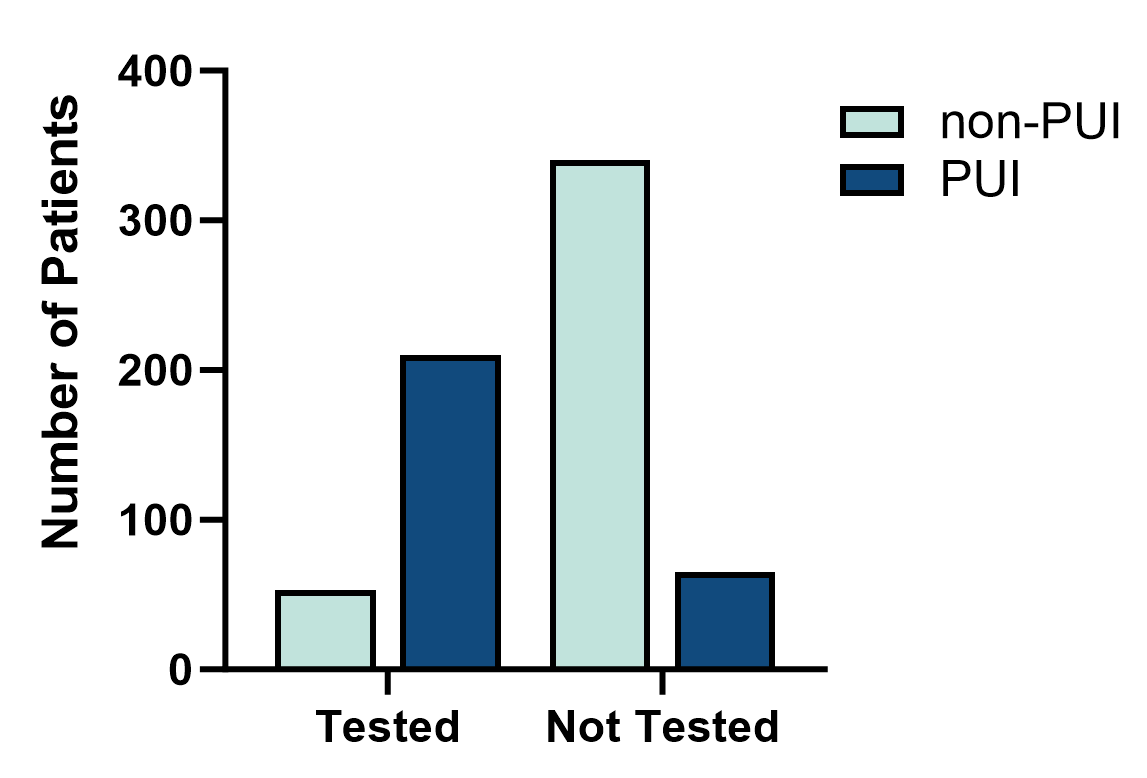


Supplemental Figure 8 – PUI vs. non-PUI number tested by polymerase chain reaction (PCR).


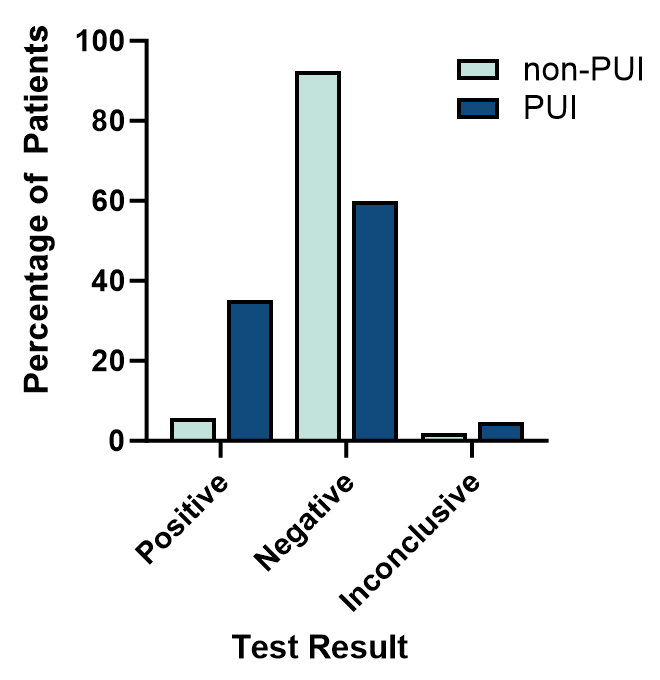


Supplemental Figure 9 – PUI vs. non-PUI number with indicated polymerase chain reaction (PCR) test result.


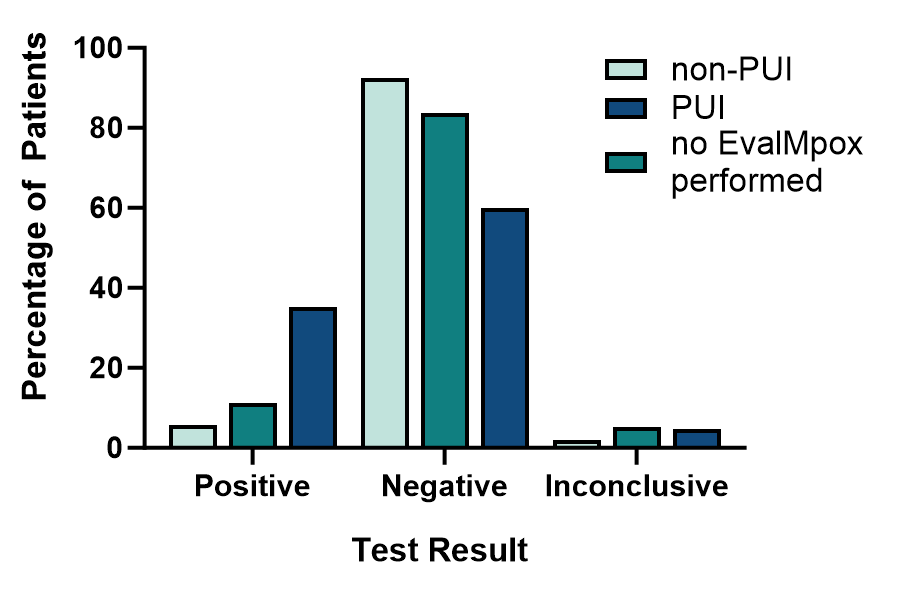


Supplemental Figure 10 - PUI vs. non-PUI number with indicated PCR test result. Additionally included in this supplemental figure is the group with PCR test results but no EvalMpox performed.
